# Blending electronic techniques with traditional teaching methods to enhance chest X-ray interpretation in medical students

**DOI:** 10.1371/journal.pone.0328159

**Published:** 2025-08-06

**Authors:** Mohsen Gholinataj Jelodar, Samaneh Mirzaei

**Affiliations:** 1 Division of Pulmonary and Critical Care Medicine, Department of Internal Medicine, Shahid Sadoughi University of Medical Sciences and Health Services, Yazd, Iran; 2 Clinical Research Development Center, Shahid Rahnemoon Hospital, School of Medicine, Shahid Sadoughi University of Medical Sciences and Health Services, Yazd, Iran; 3 Department of Health in Disaster and Emergencies, School of Public Health, Shahid Sadoughi University of Medical Sciences and Health Services, Yazd, Iran; University of Pisa, ITALY

## Abstract

**Introduction:**

Medical students must possess the skill of interpreting chest X-rays, as it is vital for accurately diagnosing patients and determining the correct treatment methods. However, there is a lack of adequate reporting on satisfactory radiology interpretation skills. This study aims to examine the impact of an electronic educational program on the ability of medical students to interpret chest radiographs.

**Method:**

The study is a quasi-experimental intervention involving a pre-test and post-test. It was conducted on 4th-year medical students from two academic hospitals in Yazd (from January 20, 2024, to April 20, 2024). The students were randomly assigned to either a control group or an intervention group. First, the skill level of the participants in both groups was assessed in chest radiographic interpretation using an electronic test. The training was carried out with two intervention(N = 40) and control groups(N = 38) through a 3-hour workshop held over two consecutive weeks. Following the initial training, the intervention group received an electronic educational package consisting of 59 chest radiographs. 15 chest X-rays and educational slides were sent to the students daily via Google Forms over two weeks. A month later, the student’s skills in interpreting chest X-rays were evaluated in both groups.

**Results:**

In area of knowledge, the mean pre-test score for the intervention group was 2.7 (± 2.3), which increased to 14.13 (± 2.66) after the intervention. In the control group, the pre-test score rose from 3.26 (± 2.44) to 6.58 (± 3.44). These results indicate a significant increase in the knowledge score for the intervention group when compared to the control group (p < 0.001). In the need for a CT scan, the mean score for the intervention group increased significantly from 1.38 (± 1.4) in the pre-test to 9.63 (± 2.82) in the post-test. In contrast, the control group showed an increase from 1.53 (± 1.17) to 4.08(± 2.82). This indicates that the intervention had a significant effect on improving the ability to detect the need for a CT scan (p < 0.001). In the area of self-confidence, the intervention group demonstrated an increase from an average score of 26.83 (±10.23) in the pre-test to 73.9 (±18.38) in the post-test. In contrast, the control group experienced an increase from 30.37 (±11.5) to 46.18 (±18.38). These findings indicate a significant improvement in self-confidence within the intervention group following the implementation of the training program (p < 0.001).

**Conclusion:**

Both traditional and blended E-learning improved students’ chest x-ray interpretation skills. However, the study found that the group receiving electronic training showed more noticeable positive changes, significantly increasing students’ self-confidence. A combination of E-learning and traditional methods significantly improves chest radiograph interpretation among young students. Early integration of E-learning for CXR interpretation in the educational curriculum is recommended.

## Introduction

Chest X-ray (CXR) is one of the most common requests from the radiology department due to its high availability, low cost, and the possibility of being easily performed at the patient’s bedside [1]. This modality is an important tool in the diagnosis and monitoring of a range of cardiopulmonary disorders, from pneumonia, heart failure, and pneumothorax to suspected lung cancer, tuberculosis, or interstitial lung diseases [2,3].

With the advancement of imaging technology, medical knowledge increasingly relies on the unique anatomical and abnormality information provided by radiology, and medical students must be able to extract relevant clinical information from radiology images [4]. The ability to interpret CXR is an important skill for medical students [5,6] however previous studies have not reported satisfactory interpretation skills in medical students [7].

It has been shown that the inability to interpret CXR leads to management errors and adverse patient outcomes [8]. Chest image interpretation is a challenging and skillful task. The great variation in patient anatomy, the wide range of abnormalities that can appear on chest images, and the diverse manifestations of different abnormalities add to the challenge [4]. The cases mentioned indicate that more effective training is needed in this vital field [9].

Electronic learning(E-learning) allows students to learn and complete their skills in a stress-free, nonjudgmental environment [6,10]. It allows more dynamic interaction and feedback between professor and student [11]. It also makes education more accessible and allows the learner to learn the content at his own pace [12]. It should be noted that radiology is suitable for electronic implementation due to the very visual nature of the content [8].

One of the current issues with radiology-focused educational materials available online is that they usually need to be standardized and specifically designed for specific competency tests [7]. However, when E-learning is designed for medical students and implemented interactively, this learning method has effectively developed the ability to interpret radiology for medical students [13].

In recent years, emphasis has been placed on education to achieve fluent behavior. In fluent behavior, two components are emphasized: accuracy and speed of action. In other words, in this method, the student is not only focused on getting the correct answer but the answer and implementation in a short and fast time are also emphasized [14]. In the study by Dunne.et all., CXR training was conducted with an emphasis on fluency, and effective results were reported in learners [15].

It is important to accurately and quickly diagnose thoracic diseases, particularly those affecting the heart and lungs. Despite various studies [16,17] on E-learning CXR training, currently, studies on teaching this important modality to medical students with an emphasis on fluency are limited, and the need for further studies in this field is felt. This study aimed to evaluate the impact of education on assisting medical students in interpreting chest radiographs effectively. It explored the potential of E-learning methods to enhance students’ interpretation of chest radiographs and develop fluent behavior in them.

## Method

This quasi-experimental study was conducted among fourth-year medical students undergoing internal medicine rotation. The study samples included medical students at the beginning of the clinical medicine course in two academic hospitals in Yazd, Iran (from January 20, 2024, to April 20, 2024). After obtaining informed consent, the participants entered the study and were divided into two control groups (teaching by lecture) and an intervention group (combined teaching by lecture and electronics). Attrition criteria included non-participation in any of the initial workshop sessions or non-participation in the E-learning program for participants in the intervention group. This criterion includes not participating in more than 20% of the daily E-learning course.

Considering the effect size value of 0.4, the level of probability of type 1 error of 5%, and the level of probability of type 2 error of 20%, the sample size in each group was equal to 37 people. Due to the possibility of dropping samples, the sample volume was increased by 10%, and finally, 40 people were considered in each group using G*Power software.

The study was conducted in two phases: designing and implementing the educational program.

### The first phase: Designing the educational program

In this study, training was aimed at increasing medical students’ skill level in chest radiography interpretation. The training was designed in two phases in this study. The first phase included training all students in both groups in the conventional educational method through seminars and PowerPoint. The educational content in the workshop includes physical characteristics of x-rays, indications for requesting CXRs, examination of the indices of the quality of the films, anatomical explanation in normal radiographs, and finally, identifying the types of abnormalities found in CXRs, including disorders in the airways, bones, heart and mediastinum, diaphragm and lung parenchyma have been involved. The educational content was prepared and presented by a pulmonologist with nine years of medical experience in pulmonology and seven years of academic training.

**Fig 1:**
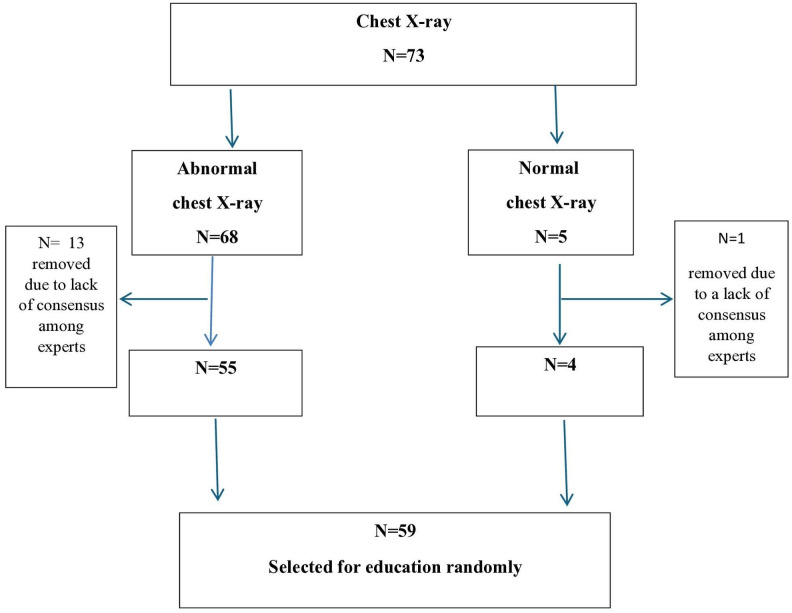
Selection of X-ray chest radiology images for education in the education design stage with experts’ opinion.

In the next phase, there is a need to design and prepare electronic education content. For this purpose, 73 chest radiographs were assessed. These radiographs were selected from open-access repositories and researchers’ collections. Three specialist doctors initially reviewed the radiographs, including two radiologists and a pulmonologist. If all three doctors agreed on the radiographs for educational purposes and reached a consensus on whether the findings were normal or abnormal and the type of abnormality present, then the radiographs were considered suitable for electronic educational use. (Fig 1)

**Fig 2:**
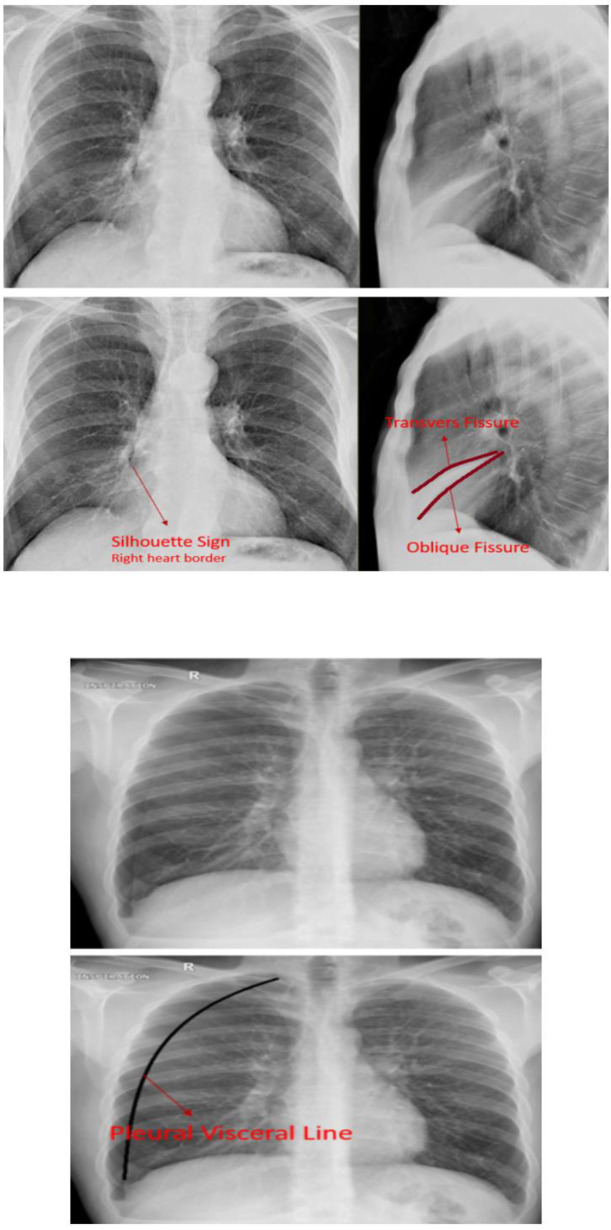
Examples of electronic content designed for student instruction, including identification of radiograph abnormalities, upper: right middle lobe atelectasis, lower: pneumothorax.

Each radiograph was placed on a single slide in PowerPoint format. The following slide displayed the correct diagnosis, with the location of any abnormalities highlighted (normal radiographs will also be included). (Fig 2)

It was essential to determine the standard duration for fluently interpreting radiographs. The researchers utilized third- and fourth-year radiology residents as experts to meet the required response time. Five residents approved by the radiology department were involved in interpreting chest radiographs for this purpose. Twenty chest X-rays (CXRs) were submitted to the residents electronically through a Google form. They were required to interpret the images accurately and swiftly. Two residents were not considered for assessment due to an incorrect interpretation of one of the provided images. In comparison, the performance of the remaining three residents was evaluated with 100% accuracy in their interpretations. Their performance determined the average time to answer each question. In this evaluation, the average achievement of correct interpretation in each chest radiograph at the residency

level was equal to 32 seconds. By adding approximately 50% to the mentioned time, the project experts found 50 seconds suitable for the correct interpretation of CXR at the student level. Based on this, the maximum time to achieve the correct diagnosis in the daily electronic educational content is set to 50 seconds. After this period, the slide is automatically changed. The next slide, which contains 30 seconds of schematic interpretation of the case discussed in the previous slide, is entered. The criterion for achieving fluency in E-learning is defined as the learner’s ability to correctly interpret all educational content sent daily at the determined time for three consecutive days.

### Implementation of the educational program

First, students’ skill level regarding chest radiography interpretation was measured through an electronic test before the training. Students were randomly divided into intervention and control groups at the workshop’s initiation. The division was allocated to each group by choosing students from envelopes A or B. Then, the CXR interpretation training program took place in two 3-hour workshops in two consecutive weeks. The educational content considered in the workshop was made available to all students after the end of the training phase. After that, the conditions and goals of E-learning were explained to the intervention group by mentioning the details of the implementation method. In the intervention group, chest interpretation skill training was done electronically. Among the 59 radiographs approved by experts, 15 slides were randomly selected daily and sent to students. (Table 1)

**Table 1:** Radiographs approved by experts for education in the intervention group.

Diagnosis	Number	Type
**Air space opacity**	14	RUL:3 RML:3 RLL:2 LUL:2 LLL:2 Diffuse:2
**Mass mediastina**	4	Anterior:2 Posterior:2
**Hyperinflation**	3	–
**Lung collapse**	2	–
**Lobular atelectasis**	12	RUL:3 RML:3 RLL:2 LUL:2 LLL:2
**Pleural effusion**	7	Massive PE:2 loculated PE:2 Unilateral PE:2 Bilateral PE:1
**Pneumothorax**	3	–
**Aortic aneurism**	2	–
**Pneumomediastinum**	2	–
**Pneumoperitoneum**	2	–
**Mass lesion, Cavitary lesion & abscess formation**	4	–
**Normal**	4	–

**RUL: *Right upper lobe*; RML: Right middle lobe; RLL: Right lower lobe; LUL: left upper lobe*; LLL:* left lower lobe; PE: pleural effusion.**

E-learning content was sent every day from 4 pm to 11 pm. The educational program form was designed using Google form so the participants could record their diagnosis under each slide. After recording the answer by the participant in the next slide, the correct diagnosis and abnormality in CXR, if any, were shown schematically along with details.

The duration of responding to each slide was 50 seconds, and the duration of the correct answer slide was 30 seconds. As time passed, the slides were changed one by one. The number of correct and incorrect answers was determined daily. This educational package was implemented daily for two weeks in the intervention group. Daily reminders were also sent to the participants through Google Calendar. In addition, the progress of the response answers was sent to the participants every three days, and the progress in the correct interpretation of the radiographs was checked for them. If there was no improvement or progress, they were contacted to review the educational content again. Every

three days, we evaluated the results of the overall interpretation of the presented slides and identified the abnormalities with the most incorrect interpretations among the students. Abnormalities were considered wrong answers from at least 30% of students in that radiograph. We then sent the abnormalities shown schematically on radiographs and an audio file to the intervention group formed on the social network. This was done to emphasize the key points for correct interpretation further.

### Evaluation of the educational program

Before and after the intervention, students’ skill in interpreting chest radiographs was measured in two groups, one month apart.

It is worth mentioning that for the electronic test before and after the study, 20 chest radiographs were selected in common cases required for the clinical work of general practitioners, which were approved and fully agreed upon by the study experts. (Table 2)

**Table 2:** Radiographs selected to evaluate students before and after the intervention in the two study groups.

Number of chest X-rays	Diagnosis
1	Left lung collapse
2	Pneumoperitoneum
3	Aortic aneurysm
4	Pneumonia of the upper lobe of the right lung
5	Pneumonia of the middle lobe of the right lung
6	Lung abscess
7	Lung Hyperinflation
8	Pneumonia of the upper lobe of the left lung
9	Pneumothorax
10	Normal
11	Anterior superior mediastinal mass
12	Foreign body aspiration
13	Pneumomediastinum
14	Pulmonary edema
15	Atelectasis of the middle lobe of the right lung
16	Massive left pleural effusion
17	lingula consolidation
18	Atelectasis of the upper lobe of the right lung
19	Pleural effusion of the left lung
20	Normal

All students were asked to determine whether the chest X-ray was normal or abnormal and express their diagnosis. Is there a need to perform a chest computed tomography (CT) scan for further investigation? Also, a 5-point Likert scale will be used to indicate the confidence level in the diagnosis. The scale is as follows: 1 for “I am not sure,” 2 for “I am less sure,” 3 for “medium,” 4 for “very sure,” and 5 for “completely sure.” Therefore, the maximum “overall certainty” for the 20 presented radiographs was 100 points. At this stage, people were not allowed to consult external sources. The response time for each question was 50 seconds. The initial electronic test was conducted in person before the start of the first session of the CXR interpretation workshop, and the final test was also conducted in person and at the previously announced time by sending a Google form from all participants.

To collect the data in this study, first, the demographic profile form, which includes age and gender, and the questionnaire to measure the students’ skill level were used. Before and one month after the electronic training session, the skill level in chest X-ray interpretation was measured using the prepared electronic file in both groups.

### Ethics approved and consent to participate

Verbal informed consent was obtained from all participants before their enrollment in the study. Trained research staff conducted the consent process, providing participants with detailed information about the study’s purpose, procedures, potential risks and benefits, and their rights to voluntarily participate and withdraw at any time without consequence. The verbal consent and its confirmation were recorded in the research log by the assigned investigator.

This study was approved by the Clinical Research Development Center at Shahid Rahnemoon Hospital in Yazd and received an ethical code (IR.SSU.SRH.REC.1402.023) from the Research ethics committees of Yazd Shahid Dr. Rahnemoun Hospital -Shahid Sadoughi University of Medical Sciences. The authors fully adhered to ethical standards, ensuring that there was no data fabrication, double publication, or plagiarism. All methods were carried out in accordance with the relevant guidelines and regulations.

Data analysis was done using SPSS version 26 software. Descriptive statistics included absolute and relative frequency, mean, and standard deviation. The inferential statistics used included paired t-test, analysis of variance, and chi-square with a 95% confidence interval.

## Results

In this study, 80 medical students were divided into two intervention groups, with 40 people in each group. Two people from the control group were excluded from the study due to incomplete attendance at the training workshops. Data analysis was conducted on 38 people in the control group and 40 in the intervention group. The chi-square and t-test results showed no significant differences between the two groups regarding age and gender. The mean ages of the control and intervention groups were 21.18 ± 0.56 and 21.28 ± 0.64 years, respectively.

**Fig 3:**
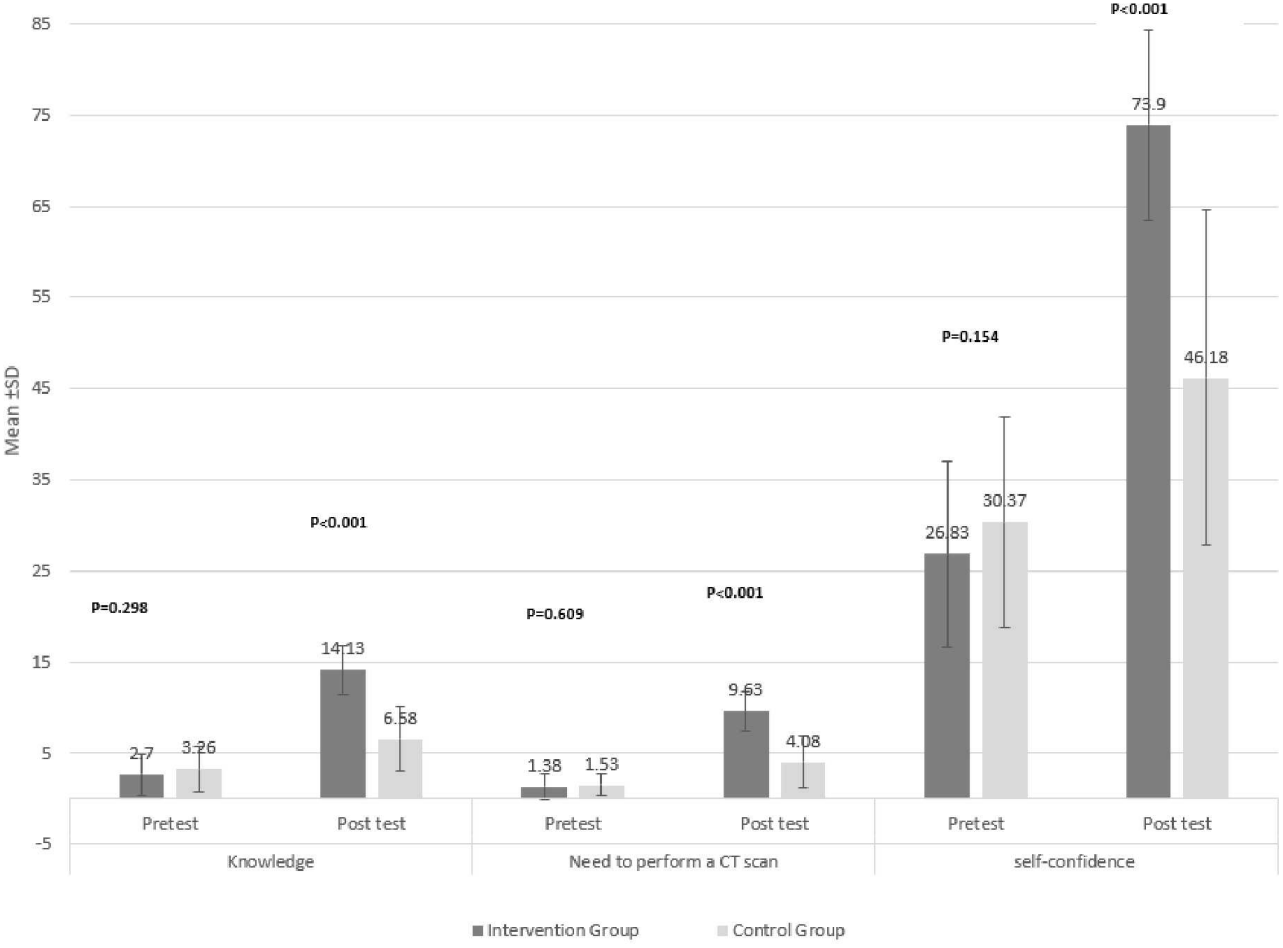
Comparison of the mean and standard deviation of the level of knowledge, diagnosis of the need to perform a CT scan, and students’ self-confidence in chest interpretation in the studied groups.

According to the findings of the study, the average scores in the field of knowledge, the need to perform CT scans, and the self-confidence of medical students before the study in the studied groups with the independent t-test did not have a statistically significant difference, but after the study (one month later) was reported to be significant. (Fig 3)

In area of knowledge, the mean pre-test score for the intervention group was 2.7 (± 2.3), which increased to 14.13 (± 2.66) after the intervention. In the control group, the pre-test score rose from 3.26 (± 2.44) to 6.58 (± 3.44). These results indicate a significant increase in the knowledge score for the intervention group when compared to the control group (p < 0.001). In the need for a CT scan, the mean score for the intervention group increased significantly from 1.38 (± 1.4) in the pre-test to 9.63 (± 2.82) in the post-test. In contrast, the control group showed an increase from 1.53 (± 1.17) to 4.08(± 2.82). This indicates that the intervention had a significant effect on improving the ability to detect the need for a CT scan (p < 0.001). In the area of self-confidence, the intervention group demonstrated an increase from an average score of 26.83 (±10.23) in the pre-test to 73.9 (±18.38) in the post-test. In contrast, the control group experienced an increase from 30.37 (±11.5) to 46.18 (±18.38). These findings indicate a significant improvement in self-confidence within the intervention group following the implementation of the training program (p < 0.001). Also, the comparison before and after the study in each of the groups independently showed that the evaluated indicators have improved significantly in each of the groups(p < 0.001) (Fig 3)

**Fig 4:**
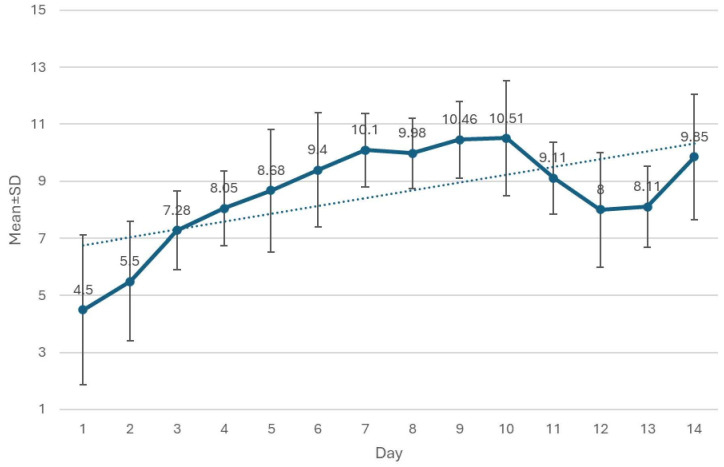
The average daily scores of CXR interpretation in the intervention group.

The interpretation scores of medical students in the electronic education group were evaluated daily. The average scores of their interpretations during the two-week E-learning are shown in Fig 4.

In this study, the level of student satisfaction with the training course held in two groups was investigated, which showed that the students in the E-learning group were significantly more satisfied with the training course. (Table 3)

**Table 3:** The opinions of the participating students in the two groups on how to hold the training course.

	Min-max	group	Mean±SD	T	p-value
Was the duration of the workshop appropriate?	1-5	control	3.31 ± 1.17	−6.58	<0.001
		intervention	4.70 ± 0.56		
Were the objectives of the workshop well stated?	1-5	control	3.84 ± 0.84	−5.65	<0.001
		intervention	4.72 ± 0.45		
Were the theoretical points explained well?	1-5	control	3.97 ± 0.82	−4.60	<0.001
		intervention	4.70 ± 0.51		
Were the deficiencies in the radiographic interpretation of the work resolved by the instructor?	1-5	control	3.88 ± 0.83	−3.42	<0.001
		intervention	4.50 ± 0.71		
Did the workshop instructor have scientific and practical mastery in the discussed field?	1-5	control	4.22 ± 0.70	−4.64	<0.001
		intervention	4.83 ± 0.38		
Did the materials presented in the workshop meet your expectations?	1-5	control	3.88 ± .090	−5.40	<0.001
		intervention	4.78 ± 0.48		
How much do you recommend participating in the workshop to your friends?	1-5	control	4.25 ± 0.76	−4.16	<0.001
		intervention	4.83 ± 0.38		
How satisfied were you with the workshop?	1-5	control	3.94 ± 1.04	−4.97	<0.001
		intervention	4.83 ± 0.38		
total	5-40	control	31.26 ± 5.86	−6.44	<0.001
		intervention	37.87 ± 2.70		

## Discussion

This study investigates the effect of the training program by combining the conventional method with short-term electronic training. Considering that CXR interpretation is a vital skill for medical graduate students that can guide them toward more accurate diagnosis and treatment of patients, identifying an effective method for developing competence in radiology interpretation is vital for medical students [4].

Based on the study’s findings, E-learning has significantly increased the knowledge of chest radiography interpretation, which shows the effect of short-term electronic training on increasing the knowledge of radiology interpretation. In this study, E-learning has increased students’ ability to correctly diagnose the need to request a CT scan to confirm the diagnosis and provide treatment in the next stages, as well as increase the student’s self-confidence to diagnose and interpret chest X-rays. Examining the two groups separately has shown that although both education methods have increased knowledge, the ability to recognize the need for CT and students’ self-confidence plays an effective role. However, the grades are significantly higher in the electronic teaching method than in the lecture method. Considering the current era and the increase in the use of virtual space, turning to electronic education can be considered more in educational systems. According to the study by Wentzell et al. (2018), electronic training in chest interpretation has increased the knowledge of chest radiographic interpretation in the middle and after the intervention compared to before [18]. In the study of Keijzersa and Sithirasenana (2012), it was also shown that teaching CT interpretation to doctors showed that lecture-style training could not affect increasing the knowledge scores, diagnosis, or overall scores of the doctors participating in the study [19]. A combination of traditional and electronic learning has also been effective in interpreting the radiographs of medical students, as Salajegheh et al. (2016) showed in their study. In other studies, it has been shown that the final-year medical students who were trained in the initial years had higher scores in the interpretation of chest radiographs [18,20]. The ability to interpret X-rays is a critical skill for graduate medical students that guides physicians toward accurate patient diagnosis and treatment, and E-learning is an effective way to develop radiology interpretation competency for medical students [4]. The findings of these studies were in line with the current study’s findings.

In this study, the speed of the students’ operation was also considered in addition to strengthening the correct interpretation of chest X-rays. Therefore, the training and evaluations carried out in this study were based on correct faults over a limited time. In interventions with the goal of comprehensive behavior fluency, one should achieve a predetermined level of skill in a specific time frame [21]. Based on the study of radiology residents, we considered the time required for the correct interpretation of each radiograph to equal fifty seconds for general medicine students. In this method, both the continuous measurement of behavior and its feedback are given to the learner [22]. The beneficial results of using this method have been shown in various medical education studies [23–25]. In our study, none of the learners achieved the secondary goal, which was to develop fluent behavior in CXR interpretation. Despite a significant improvement in the initial study objectives after two weeks of E-learning, creating fluency was not achieved. It seems that achieving this valuable point requires training over a longer period.

E-learning can be an independent tool for increasing medical students’ knowledge of interpreting chest radiography. Medical students can also use it before starting their clinical placement, which may give them greater confidence in examining patients and interpreting chest radiographs on the wards [3,17].

Based on the survey, medical students had a positive view of E-learning and felt that it helped them learn how to interpret chest radiographs and would recommend it to other students. Other studies have shown that students were satisfied with the E-learning resource [17,26]. The study by Wentzell et al. (2018) also showed that out of 118 students who provided feedback after the intervention, 102 (86.4%) would recommend the electronic resource to their colleagues to improve their interpretation skills. This study shows that early exposure to E-learning radiology modules in general medical school curricula is beneficial [18].

The positive feedback from students, the improvement in interpretation skills, and the increased level of confidence demonstrated by students indicate that most E-learning radiology courses can be very beneficial in the medical school curriculum in the years before entry into clinical practice, especially in the clinical setting. Be useful for current and challenging learning where there is an increasing need for digital resources. Further studies are needed to fully investigate the perceptions of medical students and faculty regarding radiology teaching methods and to evaluate the long-term retention of radiology interpretation skills after using an E-learning X-ray interpretation module. Nevertheless, the E-learning intervention demonstrated measurable improvements in students’ skills in interpreting chest X-rays (CXR). The inclusion of interactive elements, such as question-and-answer sessions and both individual and group feedback, enhanced the educational impact of the program. While the intervention showed an immediate positive effect, the study did not evaluate long-term knowledge retention or the students’ ability to interpret X-rays during their clinical clerkships. A long-term follow-up would therefore strengthen the validity and impact of the findings.

The main strength of our study was the E-learning-based training with an emphasis on improving fluency in chest X-ray interpretation. Another strength of the study was the focus on training medical students at the beginning of their clinical careers.

We encountered some limitations in this study. Contamination of the two study groups has been our concern since the beginning of the study design, so we have used the following strategies to reduce this possible situation and prevent it.

1. Time limit for sending educational files via Google Form and deactivation of the relevant link after a daily time period
2. Selection of the control and intervention groups from two separate teaching hospitals
3. Emphasis on the intervention group students to not sharing educational files with the other group during the study

Obviously, we have not been able to fully control the aforementioned limitation and we continue to consider this as our main limitation.

another limitation in our study, is that educational intervention was restrict to only two weeks. This study time limit was somehow forced upon us due to constraints in maintaining long-term access to participants and concerns about attrition.

## Conclusion

The study’s findings highlight the importance of early inclusion of chest radiology training and interpretation in the medical school curriculum. Increasing students’ exposure to radiology images during the period of clinical course initiation can improve their diagnostic abilities and confidence. A combination of E-learning and conventional methods causes significant chest radiographic interpretation among young students. Early use of E-learning for CXR interpretation in the educational curriculum is recommended.
